# Image-guidance in endoscopic pituitary surgery: an in-silico study of errors involved in tracker-based techniques

**DOI:** 10.3389/fsurg.2023.1222859

**Published:** 2023-09-15

**Authors:** Aure Enkaoua, Mobarakol Islam, João Ramalhinho, Thomas Dowrick, James Booker, Danyal Z. Khan, Hani J. Marcus, Matthew J. Clarkson

**Affiliations:** ^^1^^ Wellcome/EPSRC Centre for Interventional and Surgical Sciences, University College London, London, UK; ^^2^^ Division of Neurosurgery, National Hospital for Neurology and Neurosurgery, London, UK

**Keywords:** augmented reality, pituitary surgery, computer-assisted surgery, tracking, neurosurgery

## Abstract

**Background:**

Endoscopic endonasal surgery is an established minimally invasive technique for resecting pituitary adenomas. However, understanding orientation and identifying critical neurovascular structures in this anatomically dense region can be challenging. In clinical practice, commercial navigation systems use a tracked pointer for guidance. Augmented Reality (AR) is an emerging technology used for surgical guidance. It can be tracker based or vision based, but neither is widely used in pituitary surgery.

**Methods:**

This pre-clinical study aims to assess the accuracy of tracker-based navigation systems, including those that allow for AR. Two setups were used to conduct simulations: (1) the standard pointer setup, tracked by an infrared camera; and (2) the endoscope setup that allows for AR, using reflective markers on the end of the endoscope, tracked by infrared cameras. The error sources were estimated by calculating the Euclidean distance between a point’s true location and the point’s location after passing it through the noisy system. A phantom study was then conducted to verify the in-silico simulation results and show a working example of image-based navigation errors in current methodologies.

**Results:**

The errors of the tracked pointer and tracked endoscope simulations were 1.7 and 2.5 mm respectively. The phantom study showed errors of 2.14 and 3.21 mm for the tracked pointer and tracked endoscope setups respectively.

**Discussion:**

In pituitary surgery, precise neighboring structure identification is crucial for success. However, our simulations reveal that the errors of tracked approaches were too large to meet the fine error margins required for pituitary surgery. In order to achieve the required accuracy, we would need much more accurate tracking, better calibration and improved registration techniques.

## 1. Introduction

The pituitary gland is situated within an exceptionally dense anatomical region, surrounded by critical neurovascular structures such as the optic nerves and internal carotid arteries (1). There is significant anatomical variation between patients, and pituitary tumours often distort this complex anatomy, making safe recognition and avoidance of critical structures difficult during surgery (2). The current gold standard surgical technique for the resection of pituitary adenomas is through a transsphenoidal approach (3). The endoscopic endonasal transsphenoidal approach allows for excellent wide-angle visualisation, but almost always relies on a monocular endoscopic camera, resulting in limited depth perception, which can further impair appreciation of critical structures (4).

Surgical navigation systems are established adjuncts used to support intra-operative orientation and navigation. The most used navigation tool is a tracked pointer, where a set of reflective markers are placed at the top of the pointer. An infrared (IR) camera tracks the markers, allowing the location of the pointer’s tip to be visualised on the pre-operative scan. This approach is cognitively demanding for the surgeon, as the surgeon must map the position of the pointer displayed on the pre-operative MRI scan onto the live endoscopic video. Moreover, it impacts the surgical workflow as the surgeon needs to repeatedly stop operating, remove their instruments, and place the probe into the operative field. Therefore, alternative techniques that remove the need for multiple displays and for manual placement of probes to regions of interest may allow for both a reduced cognitive load and an improved surgical workflow.

Augmented reality (AR) is an emerging display technology that allows structures of interest from a pre-operative MRI to be displayed directly onto the live endoscopic video. AR has already been used in several surgical procedures with varying success (5, 6). Within pituitary surgery, several research groups have previously reported the use of tracker-based AR (7, 8) but it has not been widely adopted in routine practice. A US survey that was conducted to investigate the use of intra-operative neuronavigation found that only 7% of cases used image guidance systems (9). Despite there being AR products for microscopic surgery such as the SyncAR (10), to the best of our knowledge there are no approved AR devices in endoscopic pituitary surgery. Emerging alternatives such as vision-based techniques have also been proposed although not yet widely used (11, 12).

The aim of this study was to assess the accuracy of tracker-based navigation systems, including those used for AR systems found in the research literature.

## 2. Materials and methods

### 2.1. Study overview

**Figure 1:**
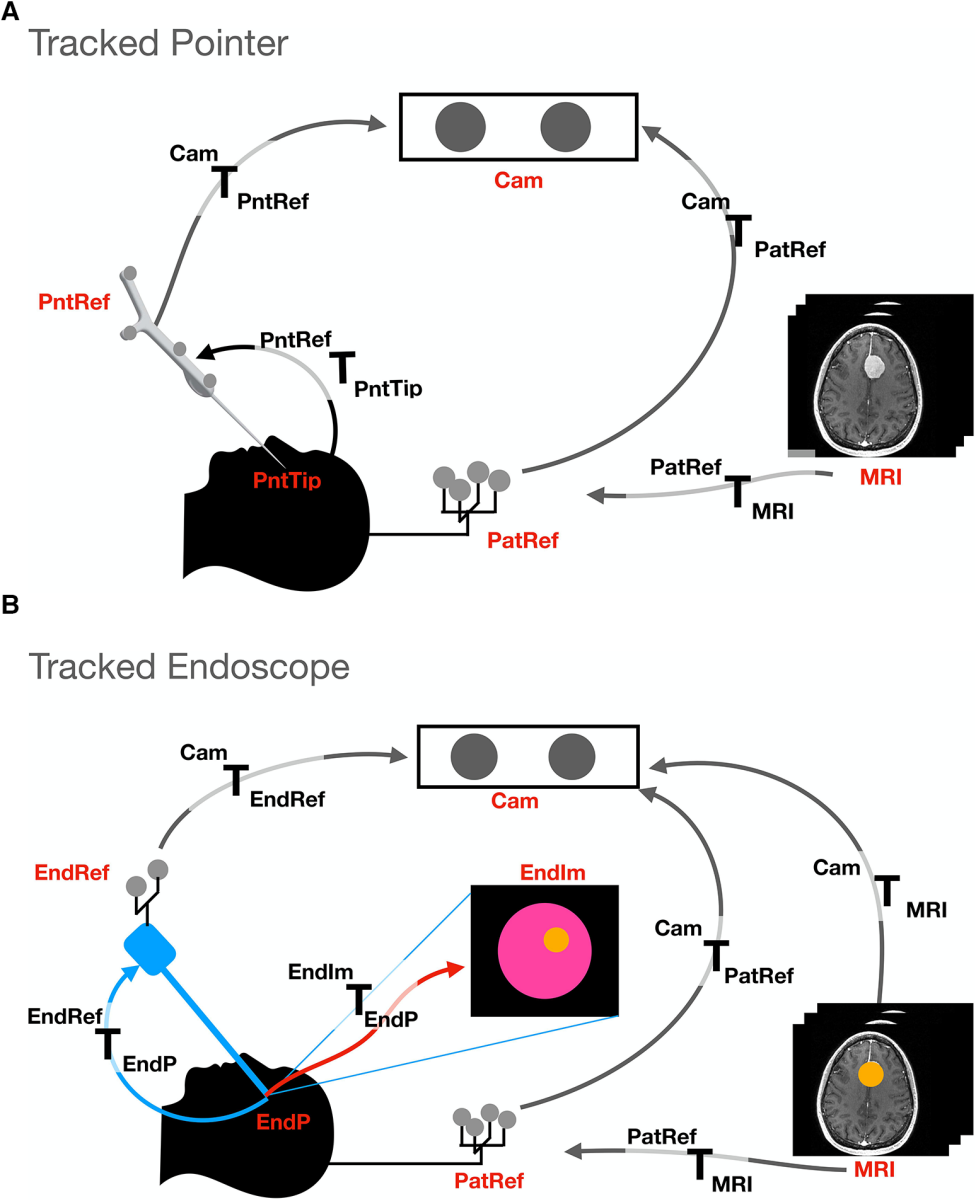
Transformations and setup involved in the two different tracking methods presented: (**A**). Tracked pointer setup, (**B**). Tracked endoscope setup. The abbreviations used stand for the different coordinate systems and are as follows- camera (Cam), pointer reference (PntRef), endoscope reference (EndRef), patient according to the mayfield clamp reference (PatRef), MRI, pointer’s tip (PntTip), endoscope tip (EndP) and endoscope video frames (EndIm).

**Figure 2:**
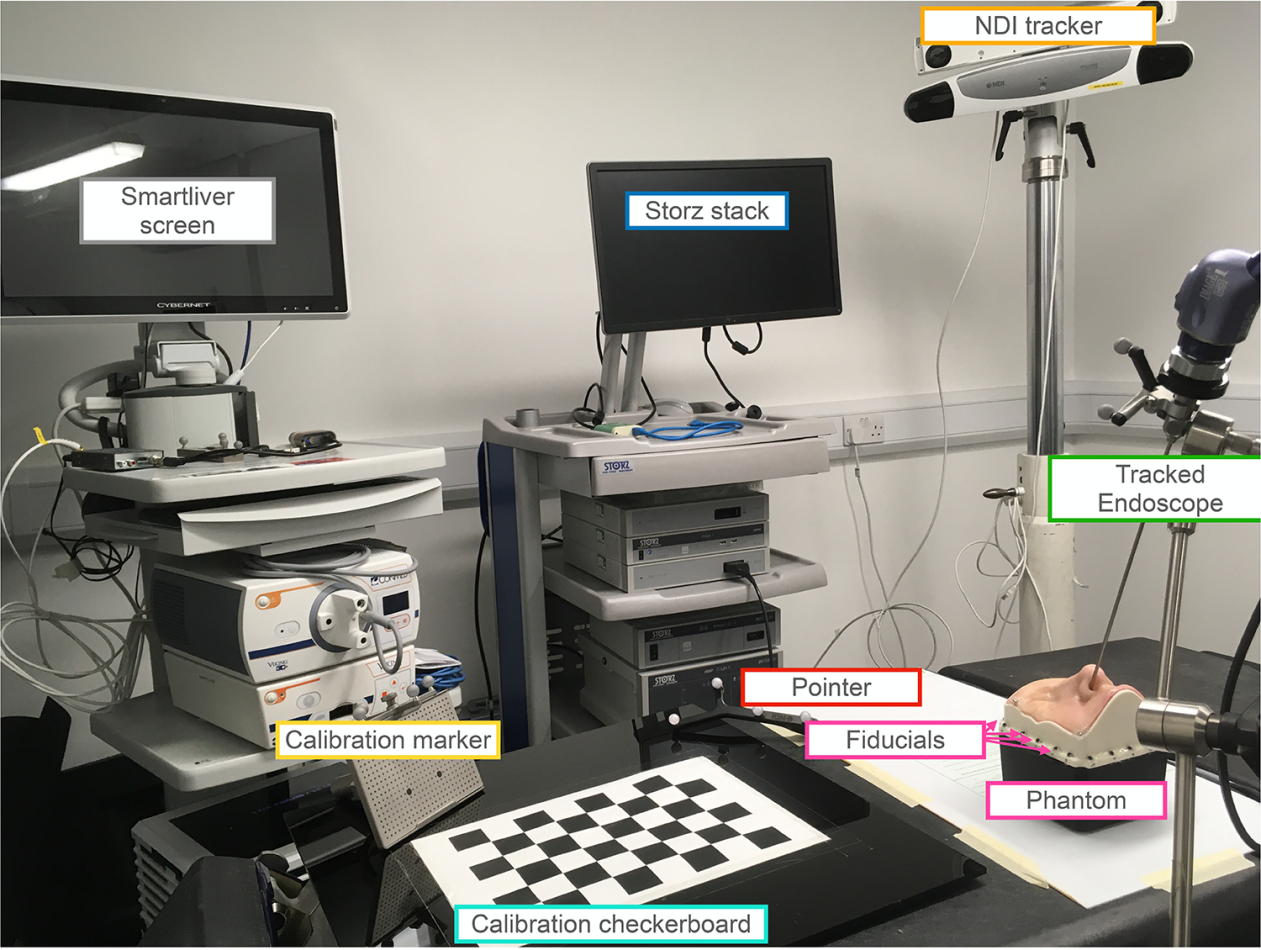
Setup for the phantom study involving both the tracked pointer and tracked endoscope setups. The endoscope is securely held in place by a clamp and positioned inside the nostril of the UpSurgeOn phantom. The phantom itself features 20 fiducials attached around its base, serving to align the CT scan and phantom to a common optical tracker space. An infrared NDI tracker is used to locate the position of the markers on the endoscope, pointer and calibration markers. The endoscope is calibrated for the AR display using the calibration checkerboard. The AR overlay is displayed on the Smartliver screen, allowing for real-time visualisation of the AR display. The Storz stack facilitates the connection of the endoscope camera and displays the endoscope’s output, providing guidance on the precise placement of the pointer tip.

We adopted a simulation-based study methodology to assess whether, under standard conditions, tracker-based guidance is sufficiently accurate to allow for guidance during pituitary adenoma resection. Simulations were developed for two different setups- a standard surgical tracked pointer setup; and a tracked endoscope setup that enabled augmented reality. The pointer and endoscope both had reflective markers, tracked by an IR camera for localisation purposes. A system overview of these setups can be seen in Figure 1 and will be described in detail in the following sections. We then conducted a phantom study to further validate our simulation results. The system setup can be seen in Figure 2.

In the navigation methods described, the shared objective is to present pre-operative imaging information to the surgeon in an intuitive fashion. This is considered as a geometrical problem where each radiological image and the physical operating theatre are described by specific coordinates which must be mapped to each other. This mapping is done via mathematical transformations and allows for information from one coordinate system to be displayed onto another. In the presented navigation methods, the IR camera can track the 3-dimensional locations of all the reflective markers. A unique grouping of reflective markers attached to a tool defines a local coordinate system. In this paper, a mathematical transform is defined as ${}^{B}T_{A}$ and maps a point from the coordinate system A to the coordinate system B. Transformations are assumed to be rigid and can therefore only be composed of rotations and translations.

In the following subsections, the study design is presented to define the chosen geometrical values used in the setup of the simulations. This is followed by the two different simulation setups. Finally, we describe how noise was simulated to investigate its effects on the system accuracy.

A phantom study was subsequently conducted to verify and demonstrate the results of the simulations and show a working example of the image-based navigation errors in pituitary surgery with current methodologies.

### 2.2. Simulations

#### 2.2.1. Study design

For the purpose of the simulations, the layout of the tracker and tracked tools are defined based on realistic estimates of the physical layout in a typical pituitary surgery environment performed at a single academic neurosurgical centre.

The simulated endoscope is based on the geometry of the Karl Storz Hopkins telescope 7230 0${}^{\circ }$ of length 180 mm. The camera projection is modelled using a pinhole model and calibrated using Zhang’s camera calibration algorithm (13, 14). The pointer used in the simulation is the NDI pointer part number 8700340 with a length of 160 mm. A diagram of the components involved in each simulation can be seen in Figure 1. For both simulations, the patient is placed in front of the IR camera at a distance of 2 m. The patient is attached to a Mayfield clamp which has an NDI reference marker part number 8700339 attached to it. The distance between the patient and the reference is simplified and estimated to be 0.3 m superior to the pituitary gland.

Using the known dimensions and relative positions of the different coordinate systems, a mathematical transform can be found. With this transform, a virtual point in one coordinate system (e.g., MRI) can be converted to another coordinate system (e.g., live endoscopic video).

When the IR cameras are localising the position of the reflective markers, there is an associated error by which the IR camera localises the markers, referred to as the volumetric accuracy (σ) (15). The value of the volumetric accuracy σ by which an IR camera can locate markers can vary depending on the design of the camera. Different models such as the NDI Polaris Vega and Vicra have σ values of 0.12 and 0.25 mm respectively.^1^${}^{,}$^2^${}^{,}$^3^

Moreover, most models have a specified working range, where outside the working range the error increases, and when outside the field of view, the device stops tracking the markers accurately. The volumetric precision quoted in this article is an error value of 0.2 mm as the average found by Koivukangas et al. (15). However, values between 0–0.5 mm were investigated and can be accessed in the Supplementary material. During this simulation, the volumetric accuracy was modelled by adding noise using a Gaussian distribution with varying standard deviation σ.

The effects of noise are then calculated quantitatively by obtaining the target registration error (TRE). TRE is defined as the Euclidean distance between a point’s true location and the same point’s location after adding noise to the measurements and propagating errors through the system (16). Therefore, in order to calculate errors of different systems, the TRE is calculated between the target point set as ground truth and the same point after being transformed with the noisy transforms. To provide a meaningful average, the process of adding noise as described above is repeated over 10,000 samples of noise.

#### 2.2.2. Simulation types

##### 2.2.2.1. Tracked pointer setup

The goal of the setup is to be able to locate the pointer’s tip on the MRI scan to improve surgeons’ orientation. A transform ${}^{MRI}T_{PntTip}$ is therefore obtained to convert a point from the pointer’s tip coordinate system to the MRI coordinate system.

**Figure 3:**
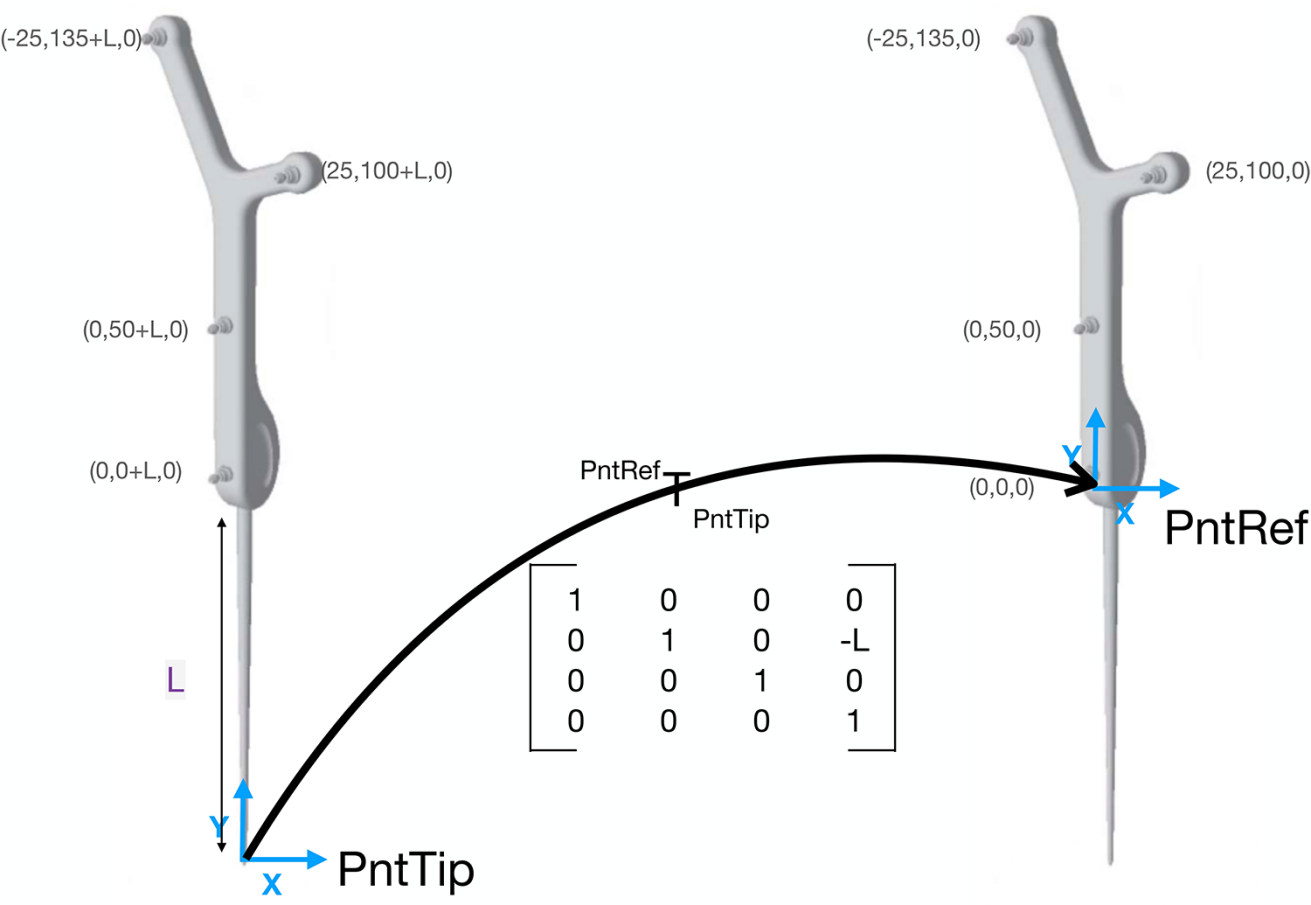
Example transformation of the tracked pointer. The coordinates of the pointer’s markers are taken from the NDI documentation sheet. The transformation ${}^{PntRef}T_{PntTip}$ is a 4×4 transformation matrix. If any point in the PntTip coordinate system is multiplied by this matrix, the point will be now in PntRef coordinates. The transformation is a simple translation in the y direction as the only difference is the length of the pointer.

Figure 1A shows the different coordinate systems of the pointer setup. The transformations between the different coordinate systems of the simulation can be deduced since the relative positions and dimensions are known by design. For example, the transform between the pointer tip and the reference is a simple translation of the pointer’s length in the y direction of the pointer’s tip coordinate system (see Figure 3). This is performed for all coordinate systems- pointer reference (PntRef) to camera (cam), camera to patient reference (PatRef) and patient reference to MRI. Once individual transformations have been obtained, it is possible to multiply any point in the pointer’s coordinate system by these transformations to obtain the same point in MRI coordinates.

##### 2.2.2.2. Tracked endoscope setup

Unlike the tracked pointer, the goal of the endoscope setup is to have an AR display of the MRI scan in endoscope coordinates. A detailed map of the involved components can be seen in Figure 1B. In this case, it is necessary to obtain ${}^{EndIm}T_{MRI}$ that will transform a point from the MRI coordinate system to each endoscopic video frame (EndIm). The relative positions of all components are known, enabling a point to be converted from the coordinate system of the MRI to the patient reference (PatRef), from PatRef to camera (cam), from cam to endoscope reference (EndRef) and from EndRef to the endoscope tip (EndP). Finally, the point is projected from a 3D point in EndP coordinates onto the 2D endoscopic video coordinate system (EndIm).

#### 2.2.3. Noise and analysis

In the two tracked setups, each transformation is prone to noise. In this section, we describe how we add realistic levels of noise onto simulated measurements and investigate what its effects are on the system accuracy.

The following errors were associated with the tracked pointer approach:

##### 2.2.3.1. Tracking noise

Since tracked tools were used in this method, there is a localisation error associated with the markers. This was modeled by adding Gaussian noise with standard deviation of 0.2 mm to the location of each marker, and recomputing the tracking transformation.

##### 2.2.3.2. Tool length effect

The effects of different pointer lengths on the tracking noise were investigated. The longer the distance between the tool’s end and the markers, the larger the associated tracking error will be as the errors magnify with increasing distance. The lengths investigated were between 100 to 300 mm in steps of 10 mm increments. The TRE associated with the tracking noise was generated for each of the lengths.

##### 2.2.3.3. Surface based registration

The total system error will also be affected by the registration transformation between the patient’s coordinate system and the MRI coordinate system. The effect of registration error was studied by adding noise to the rotation (Euler angles in ${}^{\circ }$) and translation (displacement in mm) parameters of the registration transform.

In the endoscope simulation, the same investigations on tracking error, tool length and surface-based registration error as mentioned in the pointer simulation were performed. However, there were also some additional sources of error with this setup.

##### 2.2.3.4. Hand-eye calibration error

The errors involved in the calibration of the transformation between the tracking marker on the endoscope and the camera coordinate system of the endoscope were simulated. The TRE was calculated after adding noise to the translations and Euler angles composing the camera hand-eye matrix.

##### 2.2.3.5. AR

As the endoscope setup investigates an AR display, this error can also be expressed in camera space, measured in pixels. In this simulation, noise was added to all the transformations mentioned. Once noise was added, our interest point was projected from 3D to 2D using all the noisy transformations. The TRE was then calculated between the point transformed with the gold-standard transforms and the point transformed with the noisy transforms.

### 2.3. Phantom study

The setup of the phantom experiment can be seen in Figure 2. The phantom was the UpSurgeOn BrainBox TNS model, which is used and validated for simulation training of the transsphenoidal endonasal approach (17). A CT scan of the phantom was obtained with the Medtronic O-arm CT O2 Intraoperative Imaging System.^4^

The endoscope used was the 30 cm Storz model number 27020 AA.^5^ The pointer was the same as in the simulations, of model number 8700340, the markers placed on the endoscope for tracking were the NDI reference marker part number 8700449 and the NDI polaris Vega^6^ tracked the marker locations. The Smartliver software was used (18–20) to obtain the AR view and pointer locations.

In order to match the phantom and CT to the common optical tracker space, fiducials were attached along the base of the phantom box as seen in the setup (Figure 2) and their ground truth locations were obtained manually from the CT scan. The same locations were then sampled with the tracked pointer tip and could therefore be matched using point-based registration using the procrustes algorithm (21).

**Figure 6:**
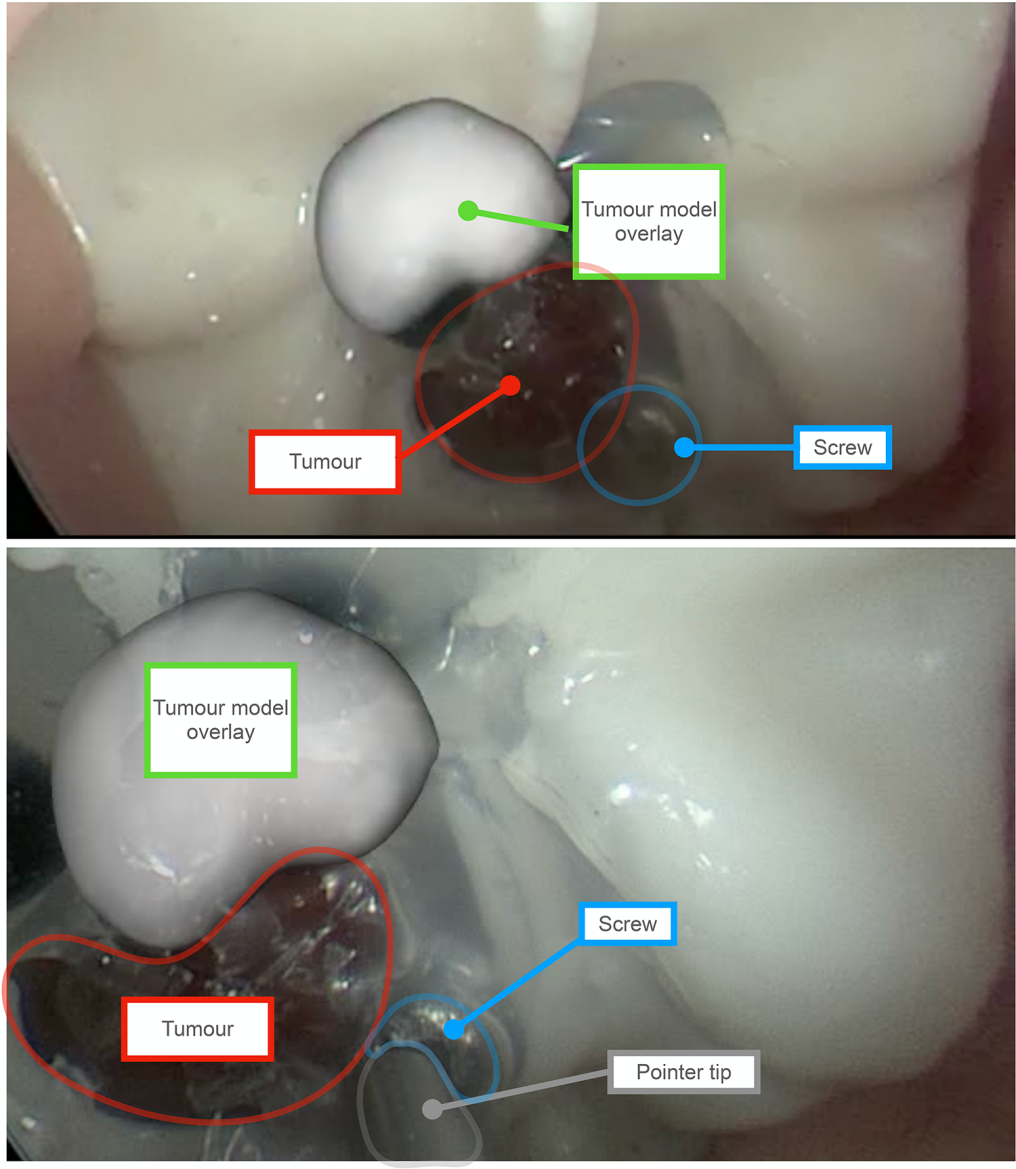
Augmented Reality (AR) overlay obtained using the tracked endoscope method. Outlines and labels are added to enhance readability. The red outline represents the phantom’s tumor, and the green label indicates the AR overlay of the tumor, showcasing an offset caused by system errors. The overlay is a 3D model of the tumour generated by manually segmenting the tumor on each slice of a CT scan of the phantom. For scale perspective, the tumor on the phantom measures approximately 8 mm in diameter. The blue outline marks the screw, which serves as the target point for the tracked pointer phantom experiment. As shown in the bottom view, the pointer tip aligns with the screw’s location. Since the precise position of the screw is known on the CT scan of the phantom, the error can be determined by calculating the difference from this location.

To replicate the pointer simulation and calculate the TRE, a screw was placed on the tumour as seen in Figure 6 to represent a target location. The pointer was then passed through the nostrill of the phantom to place its tip on the target location. Once placed on the target, the pointer tip location was recorded and averaged to represent a single point. The TRE was obtained by calculating the Euclidean Distance between the ground truth location from the CT scan, and the location obtained by the pointer when placed on the screw after being converted to CT coordinates with the point-based registration.

To replicate the tracked endoscope AR system, the surface of the tumour was segmented. This was done with the NifTK manual segmentation toolkit method slice by slice and a polygon model was then generated also using NifTK (22). The AR display was obtained using the Smartliver software (18–20) and recorded. The endoscope camera was calibrated using Zhang’s camera calibration method (14). The location of the board was tracked with an NDI reference marker part number 8700449.

To illustrate the errors caused by the tracking system, the tracked reprojection error was calculated. Since the coordinates of the chessboard corners are known, their 3D locations can be converted to the endoscope camera coordinates and projected to 2D to obtain the difference between the detected corners and the projected corners. A video of the overlay was also obtained for qualitative illustration purposes.

## 3. Results

**Figure 4:**
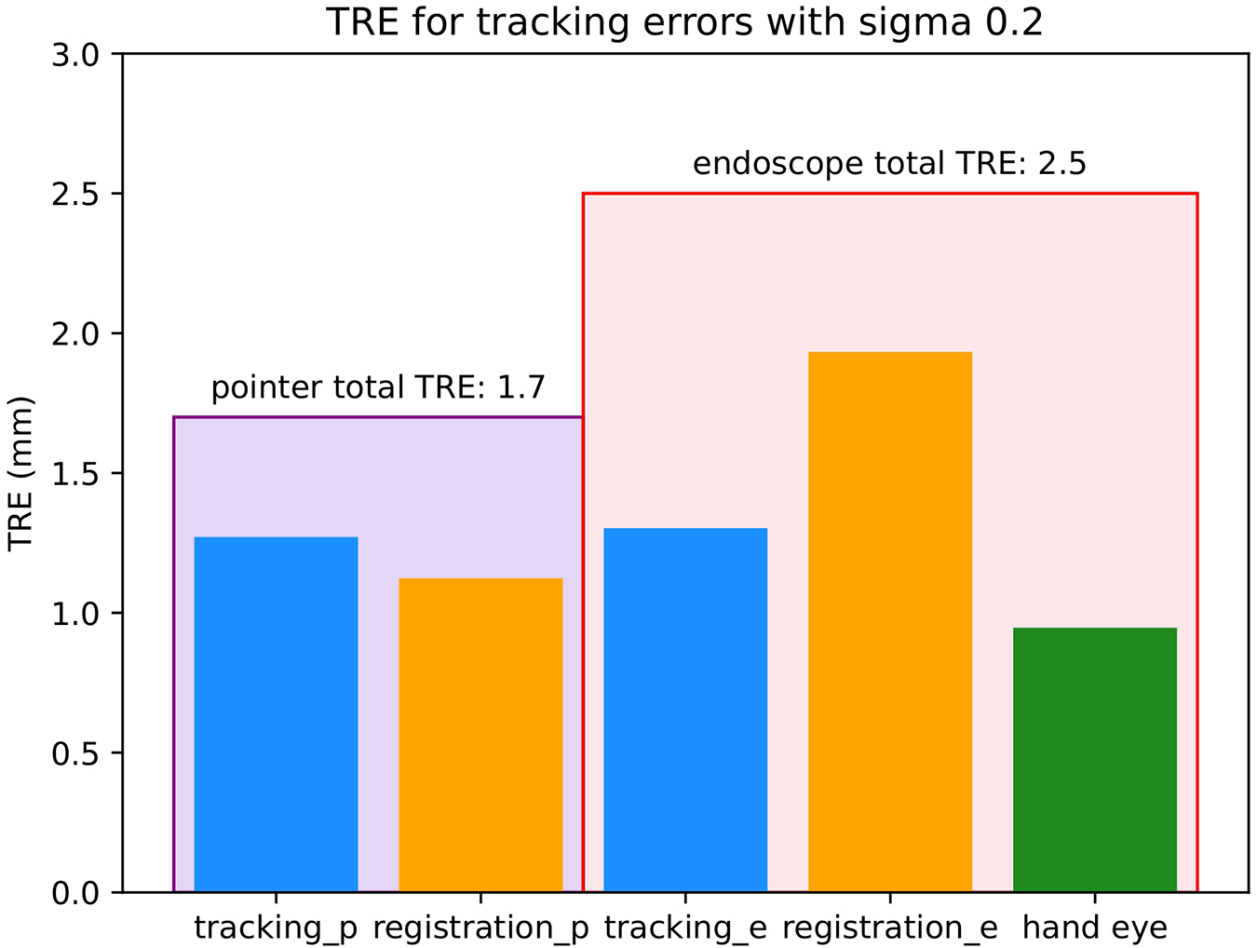
Target registration errors (TREs) involved for each of the simulations with σ 0.2 mm. All units are in mm. The large encompassing purple bar on the left represents the total TRE of the pointer simulation and the large encompassing red bar on the right represents the total TRE of the tracked endoscope simulation. Within each of these bars there are the different sources of error involved in that setup. The different sources of error are tracking (blue) which is the localisation error of the IR cameras, registration (orange) which is the surface-based registration error of MRI to patient coordinates and hand eye (green) which is the hand eye error of the endoscope.

Figure 4 shows a summary of the TREs involved in each of the simulations.

### 3.1. Simulations

#### 3.1.1. Tracked pointer

**Figure 5:**
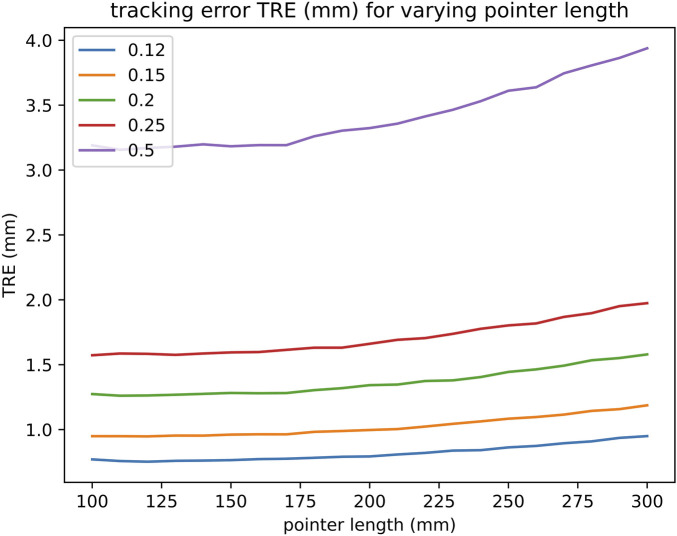
Effect of tool lengths on TRE. The different lines represent different values of σ- 0.12, 0.15, 0.20, 0.25 and 0.5 mm.

The tracking and surface-based registration errors of the pointer were simulated. As mentioned previously, the length of the pointer used in a single neurosurgical centre served as the model for this investigation. However, as pointer length can vary, the effect of tool length was also investigated on the tracking noise as seen in Figure 5. The total TRE of the pointer with σ 0.2 mm as seen in Figure 4 was composed of 2 main errors- tracking and surface-based registration errors, with values of 1.3 and 1.1 mm respectively. The TRE with all sources of error was 1.7 mm.

#### 3.1.2. Tracked endoscope

In this simulation, two different accuracies can be quoted, one for 3D and one for the 2D (AR) errors. The 3D TRE of the 180 mm endoscope as seen in Figure 4 was composed of 3 main errors- a tracking error of 1.3 mm, a surface-based registration error of 1.9 mm and a hand-eye error of 0.9 mm. The TRE in 3D with all sources of error added was 2.5 mm. The total TRE in 2D for the AR display was 29 pixels.

### 3.2. Phantom

In the phantom study, the registration used for both simulations had an error of 1.03 mm. The calibration error with Zhang’s calibration algorithm used for the tracked endoscope setup was 0.66 mm.

The TRE obtained by the tracked pointer phantom study was 2.14 mm. The tracked monocular reprojection error of the AR system was 3.21 mm, and the AR display obtained by the tracked endoscope can be seen in Figure 6

## 4. Discussion

### 4.1. Principal findings

In this simulation-based study we have, for the first time, demonstrated that tracker-based AR devices are likely to be insufficiently accurate to allow for guidance during pituitary adenoma resection. Therefore at present, the benefits of improved orientation using AR would be outweighed by the compounded errors associated with its use.

The typical size of a pituitary gland is approximately 10 mm in width and 5 mm in height (23). When the tracking camera has a σ of 0.2 mm, the error of the tracked pointer setup is 1.7 mm and that of the tracked endoscope setup is 2.5 mm. This would result in an AR display where the offset is almost half the pituitary itself, which is likely more confusing to a surgeon than helpful if used to guide resection. From the phantom study, we were able to obtain a visual representation of what the AR display may look like. As seen in Figure 6, the model of the tumour is not overlayed on the phantom’s tumour and is therefore more distracting than helpful in aiding navigation. In order to achieve a reasonable visualisation with a tracked approach, the total error of tracking, registration and any other source such as hand-eye would need to be below 1 mm. In order to achieve this accuracy, we would need much more accurate tracking, better calibration and improved registration techniques.

### 4.2. Findings in context of existing literature

Navigation systems have a long history within neurosurgery, and particularly within neuro-oncology, where they can facilitate surgical trajectory planning, resulting in shorter incisions, smaller craniotomies, and more limited brain exposure and retraction (24). Within pituitary surgery, a recent registry study reported that the use of neuronavigation was associated with improved surgical safety (25), including a reduced rate of complications such as cerebrospinal fluid (CSF) leak (26). However, the same study found that navigation was only used in approximately one in ten pituitary operations.

Limitations of current navigation systems include the need for the surgeon to mentally map the location of a point identified on the pre-operative MRI onto the live endoscopic video, and the need to repeatedly interrupt the surgery to place the pointer. This was captured by a survey that was taken on the Society of British Neurosurgeons (SBNS). Key findings were the need for “better integration with image-guidance systems (20%),” and a call for “intra-operative visualisation and improvements in neuroendoscopy (49%)” (27). One potential solution to these limitations is the use of AR, which allows for the fusion of the pre-operative MRI and live endoscope video into a single display and can do so on-demand rather than requiring a probe to be placed.

To date, there are several reports of AR systems used within pituitary surgery, dating as early as 20 years ago (8, 12, 28–43). However, no AR systems have been widely adopted despite the aforementioned stated advantages. The findings from this paper suggest that one barrier to uptake is insufficient accuracy. Although only slightly more inaccurate than standard pointer-based navigation (1.7 versus 2.5 mm), the fact that AR displays are overlaid onto the live endoscopic video make this obvious and distracting. By comparison, when using standard pointer-based techniques to identify a location on the pre-operative MRI, an experienced surgeon is more likely able to accommodate inaccuracies and interpolate the true location. The distractions posed by inaccurate AR may contribute to inattention blindness, which has also a recognised concern with such systems, and is thought to reflect cognitive overload (44, 45).

Automatic intra-operative CT scanning (iCT) is a promising technique that can be used to improve registration and can therefore boost the accuracy of the AR system (31, 46). However, its adoption faces many challenges. Firstly, it disrupts the surgical workflow as the operation has to be stopped while the image is acquired. It also increases radiation dose to both the patient and the hospital staff in the room. The head positioning needs to be altered and cannot be placed at the angle that is comfortable for operation. Finally, this method is expensive, rendering it unfeasible for low and middle-income countries.

Recently, alternatives to tracker-based navigation have emerged. The work of Mirota et al. (11, 12) introduces a vision-based system that directly matches the endoscopic video and the MRI scan without the need for a tracking system. The system functions by performing a 3D reconstruction of the endoscopic video by extracting and matching features between subsequent frames and estimating the motion between the frames. The 3D reconstructed image can then be registered with the pre-operative CT/MRI scan. Even though this paper was proposed in 2009–2011, this research has not yet reached any clinically viable solutions and there is more research to be done, especially given recent advances in deep learning techniques.

### 4.3. Strengths and limitations

The applicability of this study can be extended to various scenarios using the provided code in the Supplementary material. However, there are several points that were not accounted for in the simulation.

The primary limitation lies in how the setup estimates were derived. In order to perform the simulation, the relative positions of coordinate systems had to be determined. However, different centres may have different tools or a different setup than the one simulated. Although simulations of different tool lengths and IR camera volumetric accuracies were performed in the Supplementary material, other setup variations such as the patient-to-camera position or the relative locations of the reference coordinate systems, may influence the results as they can vary across cases. This is one of the reasons why the results of the tracked endoscope from the phantom study appeared larger than the simulations. The length of the endoscope in the simulation was 180 mm whereas the one used in the phantom experiment was 300 mm and therefore this adds to the total error of the system.

Another such point is the working range of the camera. If the tracking balls of the pointer are located outside the working range of the IR camera, the volumetric accuracy drops. Therefore, it is important to note the possibility that during a surgery, the localisation accuracy of the pointer setup changes if the surgical bed moves either too close or too far from the camera and lies outside the working range of the camera. Tracking can also stop if some of the markers are occluded.

Any tracked methods require an initial registration performed before each surgery to align the patient coordinates to the pre-operative MRI scan. Even if a highly-accurate IR camera were available in practice, the calibration errors such as the hand-eye calibration and the registration used when obtaining the transformation ${}^{PatRef}T_{MRI}$ would also need to be below 0.5 mm. This is currently unlikely in the case of surface-based registration. Other investigations comparing registration with point-based adhesive markers and surface-based registration quoted errors with surface-based registration averaging over 5 mm (47). Even though our simulations are highly analytical, the accuracy of a registration algorithm is ultimately determined by how well the surgeons perform the registration. That means that the errors will be dependent on the training provided and any time limitations the surgeon may have when performing the registration. This is also reflected in the phantom study, where the errors of the pointer and endoscope were larger than in the corresponding simulation studies by 0.41 mm and 0.71 mm respectively. The simulations we developed are simply a mathematical representation of the two setups. However, the errors are also ultimately dependent on factors that cannot be simulated such as how well the user performs registration or calibration, the model of the tracking camera, and its location in the room.

### 4.4. Conclusions and future work

The findings of this study demonstrate that a tracker-based system alone is insufficiently accurate to allow for AR in pituitary adenoma resection. To this end, future work is merited to develop either purely vision-based or hybrid vision- and tracker-based alternatives to support AR in this context.

## Data availability statement

All data generated or analysed during this study is publicly available in the **Supplementary Material**.

## Author contributions

AE: Conceptualization, Methodology, Software, Writing - Original Draft. MJC: Conceptualization, Methodology, Software, Writing - review and editing. HJM: Conceptualization, Writing - review and editing. JR, TD: Methodology, Writing - review and editing. MI, JB, DZK: Conceptualization, Writing - review and editing. All authors contributed to the article and approved the submitted version.

## Funding

This work is supported by the EPSRC-funded UCL Centre for Doctoral Training in Intelligent, Integrated Imaging in Healthcare (i4Health) [EP/S021930/1], the Wellcome/EPSRC Centre for Interventional and Surgical Sciences (WEISS) [203145Z/16/Z), and EPSRC grant [EP/W00805X/1]. Hani J Marcus is supported by WEISS and by the UCL/UCLH BRC Neuroscience. Danyal Z Khan is supported by an NIHR Academic Clinical Fellowship and a Cancer Research UK Predoctoral Fellowship. João Ramalhinho is funded by the EPSRC grant EP/T029404/1. For the purpose of open access, the author has applied a CC BY public copyright licence to any author-accepted manuscript version arising from this submission.

## Conflict of interest

The authors declare that the research was conducted in the absence of any commercial or financial relationships that could be construed as a potential conflict of interest.

## Footnotes

^1^NDI polaris vega: https://www.ndigital.com/optical-measurement-technology/polaris-vega/

^2^NDI polaris vicra: https://www.ndigital.com/optical-measurement-technology/polaris-vicra/

^3^NDI website: https://www.ndigital.com

^4^Medtronic website: https://www.medtronic.com/uk-en/index.html

^5^storz website: https://www.karlstorz.com/gb/en/index.htm

^6^NDI website: https://www.ndigital.com
