# Supplementary material for: Image-guidance in endoscopic pituitary surgery: an in-silico study of errors involved in tracker-based techniques
Source: Front Surg. 2023 Sep 15;10:1222859. doi: 10.3389/fsurg.2023.1222859 (PMC10540627; doi:10.3389/fsurg.2023.1222859)
Supplement: Supplementary file 1 [file Image1.pdf]

Supplementary Material

In this supplementary material, we provide access to the code developed for the mathematical simulations presented in the manuscript. To access the code, please follow the provided link: simulation code (1).

REFERENCES

[1]Thompson, S., Dowrick, T., Ahmad, M., Xiao, G., Koo, B., Bonmati, E., et al. (2020). Scikit-surgery: compact libraries for surgical navigation. *International journal of computer assisted radiology and surgery* 15, 1075–1084
